# Neuroprotective Effects of Sigma 1 Receptor Ligands on Motoneuron Death after Spinal Root Injury in Mice

**DOI:** 10.3390/ijms22136956

**Published:** 2021-06-28

**Authors:** Núria Gaja-Capdevila, Neus Hernández, Daniel Zamanillo, Jose Miguel Vela, Manuel Merlos, Xavier Navarro, Mireia Herrando-Grabulosa

**Affiliations:** 1 Department of Cell Biology, Physiology and Immunology, Institute of Neurosciences, Universitat Autònoma de Barcelona, 01893 Bellaterra, Spain; Nuria.Gaja@uab.cat (N.G.-C.); Neus.Solanes@uab.cat (N.H.); xavier.navarro@uab.cat (X.N.); 2 Centro de Investigación Biomédica en Red Sobre Enfermedades Neurodegenerativas (CIBERNED), 28031 Madrid, Spain; 3 Welab, Parc Científic Barcelona, 08028 Barcelona, Spain; dzamanillo@welab.barcelona (D.Z.); jvela@welab.barcelona (J.M.V.); mmerlos@welab.barcelona (M.M.); 4 Institut Guttmann Hospital de Neurorehabilitació, 08916 Badalona, Spain

**Keywords:** sigma-1 receptor, spinal root injury, motoneuron death, endoplasmic reticulum stress

## Abstract

Loss of motor neurons (MNs) after spinal root injury is a drawback limiting the recovery after palliative surgery by nerve or muscle transfers. Research based on preventing MN death is a hallmark to improve the perspectives of recovery following severe nerve injuries. Sigma-1 receptor (Sig-1R) is a protein highly expressed in MNs, proposed as neuroprotective target for ameliorating MN degenerative conditions. Here, we used a model of L4–L5 rhizotomy in adult mice to induce MN degeneration and to evaluate the neuroprotective role of Sig-1R ligands (PRE-084, SA4503 and BD1063). Lumbar spinal cord was collected at 7, 14, 28 and 42 days post-injury (dpi) for immunohistochemistry, immunofluorescence and Western blot analyses. This proximal axotomy at the immediate postganglionic level resulted in significant death, up to 40% of spinal MNs at 42 days after injury and showed markedly increased glial reactivity. Sig-1R ligands PRE-084, SA4503 and BD1063 reduced MN loss by about 20%, associated to modulation of endoplasmic reticulum stress markers IRE1α and XBP1. These pathways are Sig-1R specific since they were not produced in Sig-1R knockout mice. These findings suggest that Sig-1R is a promising target for the treatment of MN cell death after neural injuries.

## 1. Introduction

Injuries that affect the spinal roots and nerve plexuses constitute a frequent and severe pathology, mostly due to trauma caused by traffic, sport and working accidents or birth complications, resulting in partial or total loss of sensory, motor and autonomic functions in the affected limb. After axotomy, a neuronal retrograde response is rapidly initiated due to the disconnection between the neuron soma and its target, and neurons undergo changes in gene expression switching to a regenerative phenotype [1,2]. The course of this response depends on several factors such as the distance of the lesion to cell body, the type of injury, the neuron type, the age and the animal species [3,4,5]. Regarding the site of lesion, injuries of spinal roots or spinal nerves represent a proximal axotomy that results in progressive death of a significant proportion of spinal motoneurons (MNs) [3,6], and produces changes in the spinal circuits, while inducing a neuroinflammatory response [7]. Surgical intervention through nerve grafting or root reimplantation has been proposed as the only repair solution for spinal root and nerve lesions [8], but they are performed with delay from the injury. Strategies to prevent postinjury neuronal loss and to extend the time-window for surgical repair are needed to allow for subsequent axonal regeneration and eventual functional recovery. During recent years, attention has focused on several neurotrophic factors offering benefits on neuronal survival after root injuries [9,10,11,12], but their clinical use is uncertain. Less studies have reported favorable results in root avulsed animals with pharmacological treatment such as riluzole [13,14], lithium [15] or Neuroheal [16], although none has reached clinical application yet.

Sigma-1 Receptor (Sig-1R) is a transmembrane protein expressed in the central nervous system, particularly highly enriched in the endoplasmic reticulum (ER) of MNs [17]. Alterations in this receptor have been related with MN degeneration and several mutations identified on Sig-1R gene lead to different type of MN diseases, such as amyotrophy lateral sclerosis (ALS) [18,19] or distal hereditary motor neuropathy (dHMN) [20,21]. It has been reported that Sig-1R ligands (such as PRE-084, SA4503, pridopidine) have positive effects reducing MN degeneration in several experimental models. In vitro, PRE-084 exerted neuroprotection and neurite elongation activating protein kinase C in an organotypic culture of spinal cord excitotoxic damage [22], whereas SA4503 protected NSC34 cells against SOD1^G93A^-induced cell death [23]. After root avulsion in adult rats, PRE-084 administration prevented MN death by increasing GDNF expression in astrocytes [24]. Also, in a murine model of ALS, Sig-1R agonists enhanced preservation of spinal MNs and extended the lifespan of SOD1^G93A^ mice, by acting on different pathways: PRE-084 treatment via modulation of NMDA calcium influx [25], pridopidine and SA4503 through activation of the ERK pathway [23,26].

After an injury there is a calcium imbalance and misfolded proteins triggering ER stress as a self-protection mechanism for cell survival. The ER stress induces an adaptive reaction known as unfolded protein response (UPR), in which the ER-resident chaperone BiP is the main sensor. When BiP unbinds from three major effectors, inositol-requiring protein-1 alpha (IRE1α), RNA-activated protein kinase-like ER kinase (PERK) and activating transcription factor-6 alpha (ATF6), there is an intracellular signaling cascade from the ER to the nucleus. The activation of these pathways leads to changes in the gene expression profile of specific proteins and diverse cellular responses aiming to increase the capacity of the cell to restore homeostasis or trigger cell death [27]. Under ER stress or ligand stimulation, Sig-1R binds to IRE1α enhancing the survival pathway IRE1-XBP1 to restore the ER homeostasis [28]. The promotion of the IRE1/XBP1s axis by Sig-1R overexpression also reduces the levels of pro-apoptotic CHOP protein and prevents cell death [29]. Some studies have demonstrated that strategies targeting ER stress and UPR can alleviate neurodegeneration after axonal injury [30,31].

The aim of this study was to evaluate the progressive MN death caused by L4–L5 rhizotomy which mimics a postganglionic spinal root injury in adult wild-type (WT) mice. We investigated whether the administration of Sig-1R ligands (agonist and antagonist) could promote MN survival and modulation of glial reactivity after this injury, and if absence of the Sig-1R protein affects the outcome after rhizotomy in Sig-1R knockout (KO) mice. Our goal was to gain more insight about the molecular mechanisms behind injury induced MN degeneration, such as ER stress, and underlying the neuroprotective effects observed by Sig-1R ligands treatment.

## 2. Results

### 2.1. Functional Characterization of L4–L5 Rhizotomized Mice

**Figure 1:**
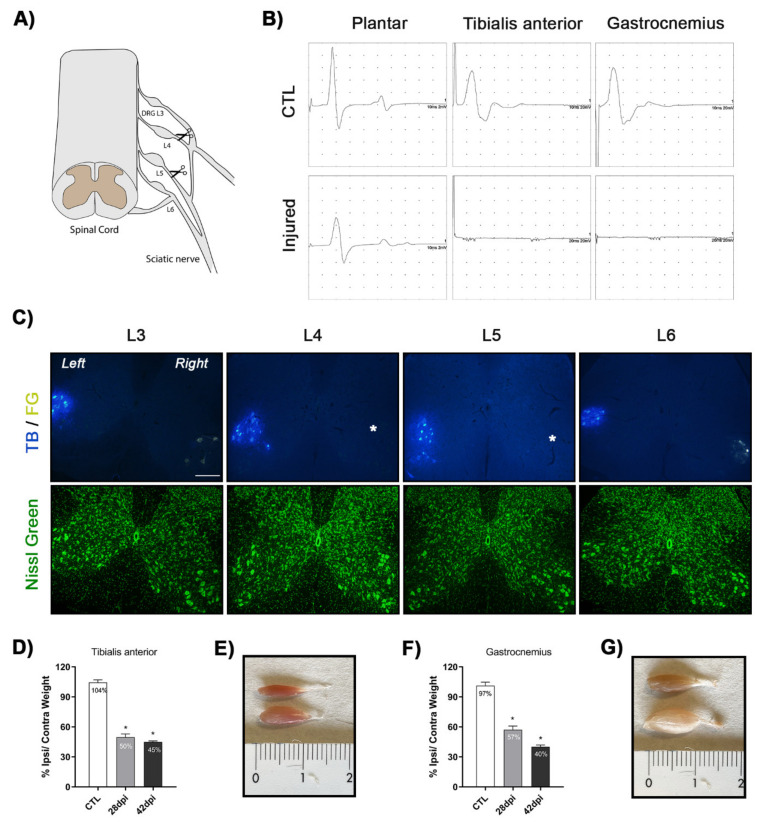
Validation of the L4–L5 rhizotomy model. (**A**) Schematic representation of the surgical procedure. After a small laminectomy, right L4–L5 roots were exposed, and a postganglionic section was made. (**B**) Motor nerve conduction tests recorded the compound muscle action potential (CMAP) in the contralateral side (CTL) and after rhizotomy (Injured). CMAPs were completely abolished in tibialis anterior (TA) and gastrocnemius (GM) muscles at 5 days post injury (dpi) whereas the plantar CMAP was reduced by two thirds. (**C**) Identification of motoneurons (MNs) by sciatic nerve retrolabeling. Representative images of lumbar spinal cord; neurons labelled with True Blue (TB, left) and FluoroGold (FG, right) counterstained with Fluoro Nissl Green. * no FG labelling. Scale bar 200 μm. (**D**–**F**) Graphs of TA and GM muscles weight ratio between ipsilateral and contralateral side at 28 and 42 dpi. Data are mean ± SEM and analyzed with ANOVA and Bonferroni post-hoc test; * *p* < 0.05 vs. CTL. *n* = 5–8 mice per group. (**E**–**G**) Representative images of TA and GM muscles in CTL and injured mice at 42 dpi, showing severe atrophy in the denervated muscles.

Nerve conduction tests showed that the compound muscle action potential (CMAP) was totally absent at 5-days post-injury (dpi) in the tibialis anterior (TA) and gastrocnemius (GM) muscles, indicating complete denervation of both muscles after L4–L5 roots section (Figure 1A,B). A reduction of the CMAP amplitude was observed in the plantar muscles in comparison with basal values since they are partially innervated by L5 and L6 ventral roots. Complete denervation of the TA and GM muscles was maintained until the end of the follow-up, confirming correct and selective lesion of L4–L5 lumbar roots. Muscles of the contralateral limb were also tested and showed CMAPs of normal amplitude (data not shown).

Injured animals presented an altered walking pattern, dragging the lesioned right hindlimb [6]. The denervated muscles suffered severe atrophy. At 28 dpi a significant reduction to 50% and 57% of the TA and GM muscles weight compared to the contralateral uninjured muscles was observed. At 42 dpi muscle atrophy progressed, with 45% (TA) and 40% (GM) weight versus the contralateral muscles (Figure 1D–G).

To identify the localization of the MN pools from L4–L5 segments, retrotracers True Blue (TB) in the left and FluoroGold (FG) in the right sciatic nerves were applied after L4–L5 rhizotomy. Spinal lumbar segments, L3 to L6, were identified by the characteristic morphology of the gray matter and comparing with the Atlas of the spinal cord [32]. TB+ MNs were observed at all the segments whereas FG+ MNs were found only at L3 and L6 segments, confirming that L4–L5 roots had been sectioned (Figure 1C).

### 2.2. MN Degeneration and Glial Reactivity Following L4–L5 Rhizotomy

**Figure 2:**
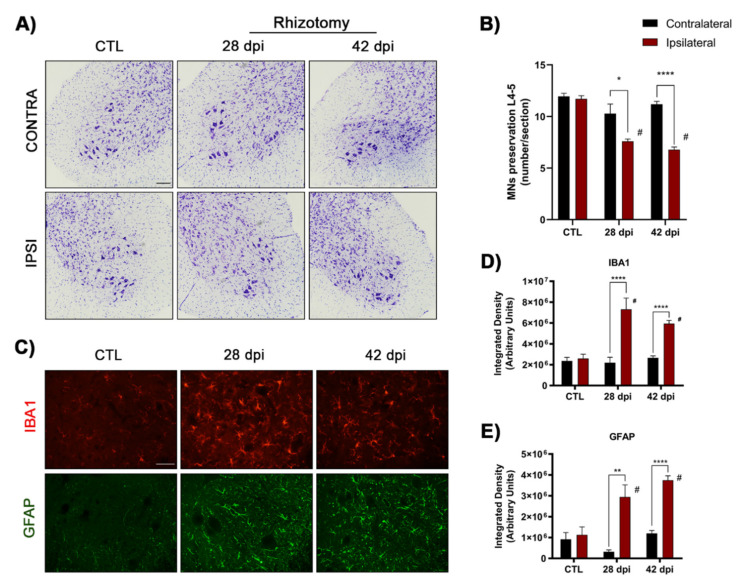
MN death and glial cell activation after rhizotomy. (**A**) Representative images of cresyl violet staining corresponding to ventral horns of L4–L5 spinal segments of the contra- and ipsilateral side to L4–L5 rhizotomy in control and in rhizotomized mice at 28 and 42 dpi. Scale bar 100 μm. (**B**) Plot of number of surviving α-MNs in L4–L5 segments, showing a decrease of MNs in the ipsilateral side to the injury; *n* = 4 female mice at 28 dpi and *n* = 14–16 animals at 42 dpi. (**C**) Images of immunolabeled microglia (Iba1) and astrocytes (GFAP) in the ipsilateral ventral horn of lumbar spinal cord. Scale bar 50 μm. (**D**,**E**) Plots of the integrated density of Iba-1 (**D**) and GFAP (**E**) immunoreactivity in the ventral horn of the spinal cord. Rhizotomy caused a significant increase of astrocyte and microglial reactivity. *n* = 3–6 mice per day and group. Data are mean ± SEM. Two-way ANOVA and Bonferroni post-hoc test; * *p* < 0.05, ** *p* < 0.01, **** *p* < 0.0001 vs. the contralateral side; # *p* < 0.05 vs. control intact mice.

The section of L4–L5 roots led to progressive atrophy and loss of MNs. MNs in the ventral horn of spinal cord sections stained with cresyl violet became progressively smaller and rounder, losing their polygonal shape, and had evidence of chromatolysis. The number of MNs in the hemispinal cord of control mice averaged 11.7 ± 0.3 (mean of number of MNs per section ± SEM), whereas after L4–L5 rhizotomy it decreased to 7.6 ± 0.2 (35% loss) at 28 dpi and 6.8 ± 0.3 (42% loss) at 42 dpi. The number of MNs in the contralateral side did not change compared to control intact cords (Figure 2A,B).

Glial reactivity was assessed by Iba1 and GFAP immunostaining in the ventral horn of lumbar spinal cords. Microglial reactivity was significantly increased in the injured side compared to control mice, being more pronounced at 28 dpi than at 42 dpi (Figure 2C,D). Similarly, a significant increase of astroglia reactivity was observed after rhizotomy similar at 28 and 42 dpi (Figure 2C–E). The contralateral side of the spinal cord did not show significant differences of glial markers in comparison with the intact control group.

### 2.3. Sig-1R Ligands Enhance Preservation of MNs after Rhizotomy

**Figure 3:**
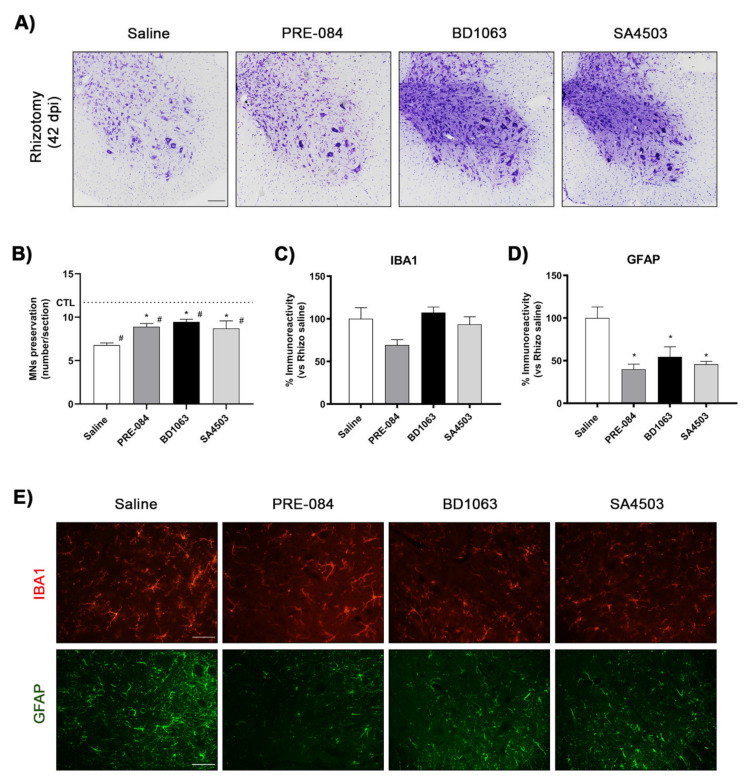
Administration of Sig-1R ligands promotes MN preservation after rhizotomy. (**A**) Representative images of ventral horns of L4–L5 spinal segments ipsilateral to rhizotomy in female mice with or without Sig-1R ligand treatment at 42 dpi. Cresyl violet stain. Scale bar 100 μm. (**B**) Plot of numbers of surviving α-MNs in L4–L5 segments, showing higher numbers of MNs in mice treated with Sig-1R ligands PRE-084, BD1063 and SA4503. Animals per group at 42 dpi: saline *n* = 11, PRE-084 *n* = 4, BD1063 = 4 and SA4503 *n* = 4. Data are mean ± SEM and analyzed with One-way ANOVA and Bonferroni multiple comparisons test. * *p* < 0.05 vs. saline rhizotomy mice at same time-point; # *p* < 0.05 vs. control mice. (**C**,**D**) Plots showing the percentage of immunoreactivity of Iba-1 (**C**) and GFAP (**D**) in the ipsilateral ventral horn of the spinal cord after rhizotomy and treatment with Sig-1R ligands. Data are mean ± SEM and analyzed with One-way ANOVA and Bonferroni multiple comparisons test. * *p* < 0.05 vs. saline rhizotomy mice. (**E**) Representative images of immunolabeling of microglia (Iba1) and astrocytes (GFAP) in the ipsilateral ventral horn of rhizotomized mice at 42 dpi. Scale bar 50 μm.

The potential neuroprotective effects of three Sig-1R ligands were evaluated in the rhizotomy model, two of them classified as agonists (PRE-084 and SA4503) and another as antagonist (BD1063) of the Sig-1R. Histological analysis showed that after 42 dpi Sig-1R ligands PRE-084 (8.9 ± 0.4 MNs per section), BD1063 (9.5 ± 0.3) and SA4503 (8.7 ± 0.9) significantly increased the number of MNs preserved between 15 to 22% more in comparison with untreated rhizotomized mice (6.8 ± 0.3) (Figure 3A,B).

Regarding glial reactivity surrounding the axotomized MNs, PRE-084 reduced by 30% microgliosis (69 ± 6% vs. saline mice) after 42 dpi although this difference was not significant (Figure 3C,E). The other two compounds did not reduce microglial reactivity compared with the saline rhyzotomy group. Astrogliosis was increased in the ipsilateral side, and it was significantly reduced in mice treated with PRE-084 (40 ± 6%), BD1063 (54 ± 12%) and SA4503 (46 ± 3%) compared with the saline group at 42 dpi. These results indicate that Sig-1R ligands can prevent MN degeneration and reduce astroglia activation after spinal root injury.

### 2.4. Influence of Sig-1R Absence in Rhizotomized Mice

**Figure 4:**
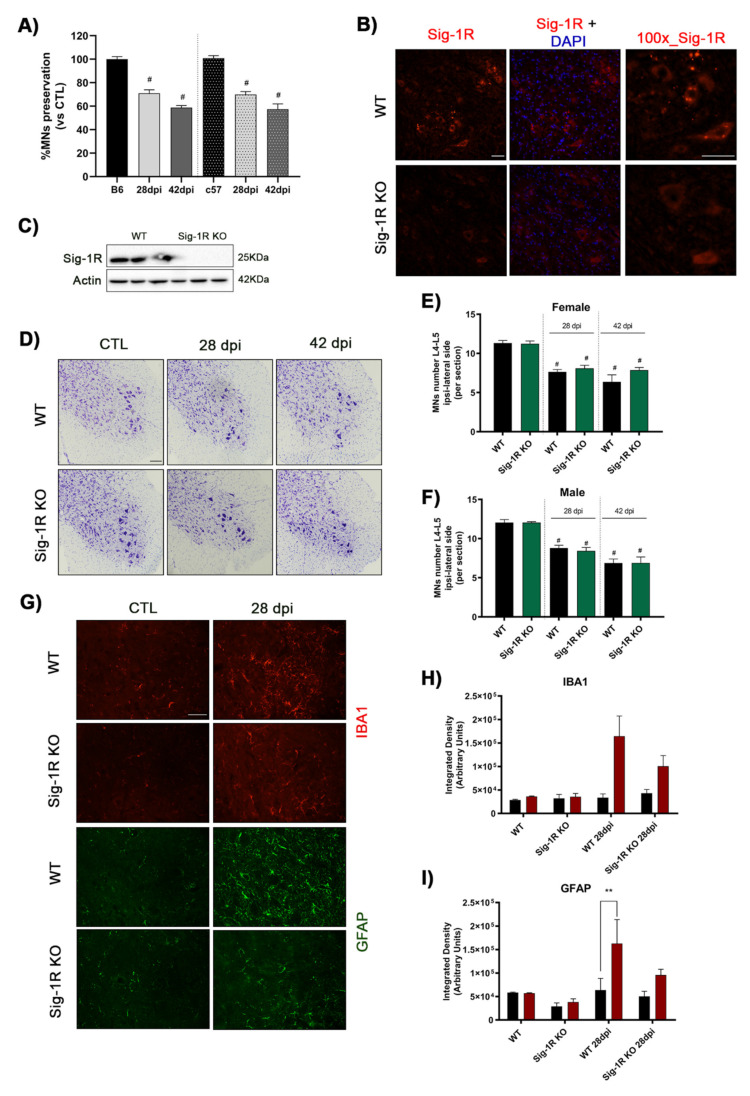
MN degeneration in Sig-1R KO mice after rhizotomy. (**A**) Plot of the percentage of surviving spinal MNs in male and female mice of the B6SJL (B6) and C57BL6 (c57) strains at 28 and 42 dpi. The same time-course of MNs loss occurred in WT mice with the two genetic backgrounds. Animals per group: B6 control *n* = 18, rhizotomy *n* = 6 at 28 dpi and *n* = 18 at 42 dpi; c57 control *n* = 16, rhizotomy *n* = 9 at 28 dpi and *n* = 9 at 42 dpi. Data are mean ± SEM with One-way ANOVA and Bonferroni multiple comparisons test. # *p* < 0.05 vs. control mice. (**B**) Representative images corresponding to Sig-1R immunolabeling in the ventral horn of spinal cord in WT and Sig-1R KO mice. There was absence of labeling in the KO mice whereas in the WT it was mainly localized in MNs. Scale bar 50 μm. (**C**) Western blot confirms the absence of Sig-1R in the spinal cord of KO mice. (**D**) Representative images of ventral horns of L4–L5 spinal segments ipsilateral to the rhizotomy in WT and Sig-1R KO mice at 28 and 42 dpi. Scale bar 100 μm. (**E**,**F**) Plots of number of surviving α-MNs in the ipsilateral side of L4–L5 segments in female (**E**) and male (**F**) mice, showing no differences between WT and Sig-1R KO mice. # *p* < 0.05 vs. uninjured mice. (**G**) Representative images of glial reactivity assessed by Iba-1 and GFAP immunolabeling. Scale bar 50 μm. (**H**,**I**) Bar graph showing the integrated density of Iba-1 (**H**) and GFAP (**I**) immunolabeling in the ventral horn of spinal cord (red: ipsilateral side; black: contralateral side). ** *p* < 0.01 vs. contralateral side.

Considering that the administration of Sig-1R ligands modulates MN survival, we investigated the consequences of the absence of this protein using transgenic KO mice with Sig-1R gene deletion. Because Sig-1R KO mice have C57BL6 background, the time course of MN death in WT mice of B6SJL and C57BL6 strains was first compared. Results showed no significant differences in the evolution of MN degeneration after L4–L5 rhizotomy between strains; the percentage of MNs loss after rhizotomy in C57BL6 WT mice was 30% and 40% at 28- and 42 dpi, respectively (Figure 4A).

In the spinal cord of WT mice, as previously described, Sig-1R was detected in MNs, specifically in clusters at the post-synaptic sites of C-terminals subsurface cistern (Figure 4B) [33,34]. This labeling was not observed in the Sig-1R KO mice. Western blot also confirmed the lack of Sig-1R protein in the KO mice (Figure 4C).

The number of spinal α-MNs in the ventral horn of L4–L5 spinal segments was the same in WT (11.3 ± 0.3 MNs per section) and Sig-1R KO (11.2 ± 0.4) adult mice (Figure 4D–F). After rhizotomy, the number of surviving MNs declined significantly, but there were no significant differences between WT and Sig-1R KO mice at the two time-points analyzed neither for female (Figure 4E) nor for male mice (Figure 4F). Sig-1R KO rhizotomized female mice had about 10% more MN preservation in comparison with injured WT mice, but this difference was not significant. In contrast, astrogliosis and microgliosis were markedly reduced in rhizotomized Sig-1R KO mice compared with injured WT mice at 28 dpi although differences did not reach significance (Figure 4G–I).

### 2.5. Activation of ER Stress after Rhizotomy

**Figure 5:**
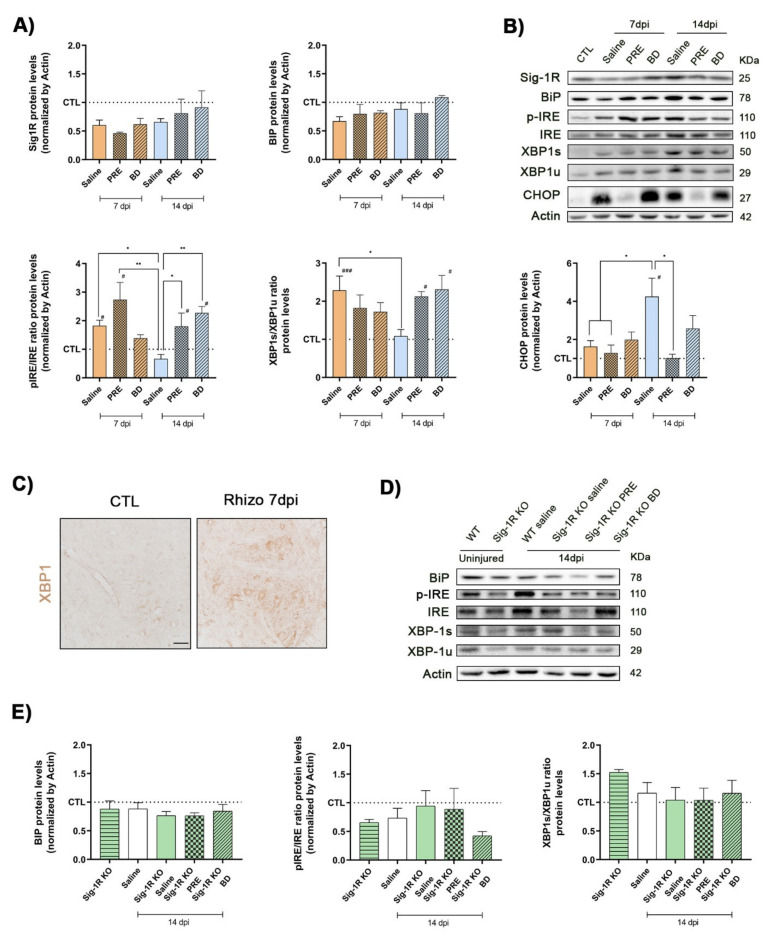
Changes in ER stress markers after rhizotomy. (**A**) Quantitative bar graphs and (**B**) immunoblots and of Sig-1R, BiP, phosphorylated IREα/IRE ratio, XBP-1s/XBP-1unspliced ratio and CHOP proteins in control (CTL) and in injured mice with or without treatment with PRE-084 and BD1063 at 7 and 14 dpi. *n* = 3–4 per group and time point. Data are mean ± SEM and analyzed with One-way ANOVA and Dunnett’s multiple comparisons test. ### *p* < 0.001 vs. CTL mice; ** *p* < 0.005, * *p* < 0.05 vs. saline 14 dpi. (**C**) Microphotographs of XBP-1 labeling in the ventral horn of spinal cord confirms the upregulation of this ER stress marker in MNs at 7 dpi. Scale bar 50 μm. (**D**) Immunoblots and (**E**) bar graphs of BiP, phosphorylated IREα/IRE ratio and XBP-1s/XBP-1unspliced ratio, in WT and Sig-1R KO mice groups at 14 dpi. *n* = 3–5 per group. One-way ANOVA and Dunnett’s post-hoc test: # *p* < 0.05 vs. CTL mice.

We then investigated whether rhizotomy induces activation of ER stress responses, similar to what was previously reported in the models of root avulsion in rats [31] and axotomy of the hypoglossal nerve in mice [35]. We found that Sig-1R protein levels in the spinal cord remained lower, although not significantly, than in uninjured control mice at 7 and 14 days after rhizotomy. Pharmacological treatments with Sig-1R ligands PRE-084 and BD1063 did not modify the protein levels of Sig-1R (Figure 5A,B). In physiological conditions Sig-1R is recruited by BiP (Grp78), which also represses the activity of the three main stress sensors, while under ER stress it is dissociated from BiP promoting the activation of downstream ER stress factors. BiP protein levels follow the pattern of Sig-1R. We did not find significant changes of BiP levels in mice after the injury, neither after the pharmacological treatments (Figure 5A). At 7 dpi after rhizotomy, in the saline group ER self-protection mechanisms were promoted through the activation of IRE1α, however at 14 dpi the ratio of activated form of IRE1α/total IRE1α declined to control levels. In contrast, Sig-1R ligands PRE-084 and BD1063 significantly increased the protective levels of activated IRE1α at 14 dpi (Figure 5A,B). IRE1α promotes the alternative spliced form of XBP1 (XBP1s), which induces the translocation of this transcriptional factor into the nucleus and the upregulation of several genes to solve ER stress. The ratio of the spliced versus unspliced forms of XBP-1 was found significantly raised at 7 days after rhizotomy in saline mice in comparison with CTL but declined at 14 days. In contrast, XBP-1s/XBP1u ratio was enhanced significantly at 14 dpi in injured mice treated with PRE-084 and BD1063. In addition, XBP-1 immunoreactivity was observed in the MNs of the ventral horn at 7 dpi compared with control samples (Figure 5C). Protein levels of CHOP were significantly increased at 14 dpi in saline injured mice. PRE-084 treatment reduced the CHOP levels at 14 dpi more efficiently than BD1063 treatment.

The ER stress markers were also analyzed in Sig-1R KO mice. There were no differences in BiP and phosphorylated IRE1α protein levels between WT and Sig-1R KO mice. Moreover, spinal root lesion did not modify the signaling axis IRE1α/XBP1s in Sig-1R KO mice, but the effects on the self-protection mechanisms by PRE-084 and BD1063 treatments were abolished (Figure 5D,E).

## 3. Discussion

Presently, there is no effective treatment for proximal spinal root or plexus injuries, other than palliative surgery through nerve or muscle transfers [8,36]. However, the death of a significant number of MNs and DRG sensory neurons after the injury presents an important drawback limiting the possibilities for recovery even after the most advanced surgical procedures [8]. Therefore, research on treatments that prevent neuronal death is necessary to improve the perspectives of recovery following severe nerve injuries. In this study we have used a model of L4–L5 rhizotomy in adult mice to induce MN degeneration and to evaluate the role of Sig-1R ligands as neuroprotective agents. This model of proximal axotomy at the immediate postganglionic level resulted in considerable death, up to 40% of spinal MNs at 42 days after the injury.

In adult animals the proximity of axotomy to the cell body significantly increases the consequences of the lesion, causing retrograde death of both spinal and cranial affected MNs. The majority of spinal MNs survive after a more distal injury, such as a sciatic nerve axotomy [37,38], but 60–80% of MNs dye following a root avulsion that traumatically detaches the axons from its entrance into the spinal cord [3,6,39]. In a model of milder lesion, a lumbar ventral root crush, it was found 25% neuronal loss at 7 days after injury, which increased to 50% at 28 days [40,41,42]. Our model of postganglionic rhizotomy is produced more distally but it avoids intraspinal surgery, and induces slower degeneration affecting 40% of MN at 42 days. Similarly, the model of hypoglossal neurotomy causes 30 to 40% MN death at 3–4 weeks post injury [35,43]. It is important to highlight that the discordance in the percentage of MN death after root injuries between some studies should also take into account differences in timing and in methods of labeling and quantifying the outcome of MNs [11].

Since survival of MNs is key to allow recovery of the motor functions lost after a neural injury and adequate repair, early and effective approaches for promoting neuroprotection after lesion are needed. We examined whether pharmacological therapy with different Sig-1R ligands could minimize the loss of spinal MNs. We found that daily treatment with PRE-084, BD1063 and SA4503 significantly increased the number of surviving MNs after L4–L5 rhizotomy. The beneficial role of Sig1-R agonist compounds in terms of neuroprotection have been previously reported on in vitro and in vivo models. In the SOD1^G93A^ mouse model of ALS neuroprotective effects with agonists PRE-084 and SA4503 were observed, mildly extending the lifespan and enhancing preservation of MNs via modulation of NMDA calcium influx and through activation of the ERK pathway [23,25]. Recently, Ionescu et al. (2019) found that pridopidine, another Sig-1R agonist, improved several cellular and histological hallmark signs of ALS [26]. PRE-084 also prevented MN death in a model of spinal root avulsion [24] and in the wobbler mouse, a spontaneous model of MN degeneration [44]. These data provide clear evidence of the interesting effects of Sig-1R agonist ligands for ameliorating neurodegenerative conditions.

Here, we demonstrated for the first time that a Sig-1R antagonist BD1063 exerted neuroprotection and reduced astroglial activation. In previous studies BD1063 was tested in coadministration with PRE-084 to demonstrate that the therapeutic action could be attributed to the activation of the Sig-1R by an agonistic compound [24,25]. In fact, in the context of Sig-1R there is controversy between the classical classification as agonists and antagonists of diverse ligand classes showing high affinity for Sig-1R [45]. Indeed, different ligands may provoke various effects, even acting opposite in the regulation of calcium homeostasis and neuronal excitability. A recent work reported that intracellular calcium shuttling can be manipulated by Sig-1R activation; the SA4503 ligand accelerated cytosolic calcium clearance after AMPA receptor activation and IP3R-mediated ER calcium release, whereas PRE-084 did not exert any significant effect on cytosolic calcium levels in SOD1^G93A^ mice cultured MNs [46]. Thus, we found promising results with different types of compounds that have high affinity to Sig-1R.

After rhizotomy, we found that Sig-1R and chaperone BiP protein levels were maintained at 7 and 14 dpi even under treatment with the Sig-1R ligands. This is in line with previous findings in which PRE-084 administration did not modify Sig-1R protein levels at 3 days after preganglionic root avulsion in rats [24]. Molecular analyses showed that L4–L5 rhizotomy induced ER stress, activating IRE1α/XBP1 pathway, as reported in other models of proximal injuries, such as root avulsion [31] and hypoglossal axotomy [35]. Upon ER stress, IRE1 undergoes dimerization and phosphorylation leading to its active endonuclease form and inducing the unconventional splicing of the mRNA encoding XBP1 with production of the XBP-1s. XBP-1s is essential for the expression of genes involved in protein folding, secretion, phospholipid biosynthesis and ER-associated protein degradation. It has been described that under ER stress or ligand stimulation, Sig-1R binds to IRE1α regulating its stability and enhancing the survival pathway IRE1-XBP1 to restore the ER homeostasis [28]. After rhizotomy we found that the IRE1/XBP1s axis is enhanced by PRE-084 and BD1063 administration, suggesting that Sig-1R ligands exert a pro-survival state by restoring ER function. Here, we also found that the activation of the IRE1/XBP1s axis is due to the modulation of Sig-1R by the ligands, since it did not occur in the Sig-1R KO mice. XBP-1s can bind to gene promoters to regulate gene expression, among others of CHOP, to restore protein homeostasis. Indeed, the ER stress induced after rhizotomy resulted in an increase of pro-apoptotic CHOP levels that would contribute to MN loss. The reduction of CHOP levels by treatment with Sig-1R ligands appeared associated to increased preservation of the spinal MNs. Similarly, in cardiomyocytes, the overexpression of Sig-1R under ER stress enhanced IRE1/XBP1s axis and reduced CHOP levels, preventing cell death [29]. Alternatively, under stress conditions such as neural injuries, Sig-1R could form cholesterol-enriched microdomains in the ER as proposed recently in vitro [47]. These changes in the ER membrane may modulate recruitment of ER proteins, such as IRE1, by forming big clusters enriched with Sig-1R and IRE1. Sig-1R modulation by administration of ligands might be influenced by this formed microdomains. However, further experiments are needed to confirm cholesterol-microdomains formation in the ER after axonal injury and possible changes upon Sig-1R stimulation.

Spinal root injury is associated with a neuroinflammatory response involving local microglia and astrocytes in the spinal cord [3]. Several reports demonstrated the important role of Sig-1R in non-neuronal cells, since in different neurodegenerative disorders the resulting glial activation was modified after treatment with a Sig-1R ligand, such as in stroke [48], pain [49] and MN diseases [24,25,44,50]. We have found in samples at 6 weeks post rhizotomy that PRE-084 treatment reduced by 30% microgliosis, whereas PRE-084, BD1063 and SA4503 administration significantly reduced astrogliosis to around 50% of untreated cords. Such more pre-eminent action on astrocytes was also described for PRE-084 treatment in the Wobbler mouse [44]. Thus, modulation of the glial response is a targeting mechanism of Sig-1R ligands, although the effects may vary depending on the compound and the type of disease. Furthermore, it is well known that non-neuronal cells express Sig-1R in the brain but only a few studies have described its expression in the spinal cord. Choi et al. (2016) found that Sig-1R immunolabeling increased in dorsal horn astrocytes following thoracic spinal cord hemisection, but not in neurons and microglia. Sig-1R protein levels also increased at day 7 post-surgery compared with control mice in the dorsal horn and normalized at 28 dpi [51]. In our study of rhizotomy, Sig-1R protein levels in spinal cord samples remained reduced at 7 and 14 dpi, although not significantly. However, it is important to note that some commercially available antibodies have poor specificity for Sig-1R, generating controversy in data reported in the literature [52].

## 4. Materials and Methods

### 4.1. Animals and Experimental Design

Adult mice (10 weeks of age) of B6SJL and C57BL6 background were maintained at the Animal Research Facility of Universitat Autònoma de Barcelona. Adult transgenic Sig-1R knockout (σ1R−/−; σ1R KO) mice (10 weeks old) with C57BL6 background, generated and characterized as previously described [53], were provided by Esteve Pharmaceuticals from the colony maintained at Envigo. Mice were kept under standard conditions of temperature (22 ± 2 °C) and 12:12 light:dark cycle with access to food and water ad libitum. Mice were maintained and handled in accordance with the guidelines of the European Union Council (Directive 2010/63/EU) and Spanish regulations on the use of laboratory animals. The experimental procedures were approved by the Ethics Committee of the Universitat Autònoma de Barcelona.

WT B6SJL female mice were submitted to L4–L5 rhizotomy and evaluated at 7, 14, 28 and 42 dpi, divided in subgroups with or without Sig1-R ligands treatment. For histological studies, male mice were also used to evaluate if there were differences in MN degeneration between sexes. The following number of animals (*n* = ♀/♂) were used per group: uninjured (CTL) (*n* = 13/5), 28 dpi saline (*n* = 3/3), 42 dpi saline (*n* = 11/7), 42 dpi PRE-084 (*n* = 4), 42 dpi SA4503 (*n* = 4) and 42 dpi BD1063 (*n* = 4). For the Sig-1R ablation study, C57BL6 male and female mice were used with the following number (*n* = ♀/♂) per group: WT uninjured (*n* = 11/6), Sig1-R KO uninjured (*n* = 5/7), WT 28 dpi (*n* = 6/3) and 42 dpi (*n* = 5/4), Sig1-R KO 28 dpi (*n* = 5/5) and 42 dpi (*n* = 8/6). For molecular analyses groups of female mice were used for obtaining samples at 7 and 14 dpi (*n* = 3–5 per treatment and day).

Intraperitoneal administration of three Sig-1R ligands, two classically considered as agonists, PRE-084 (0.25 mg/kg, TOCRIS, Bristol, UK) and SA4503 (1 mg/kg, TOCRIS, Bristol, UK) and an antagonist BD1063 (5 mg/kg, TOCRIS, Bristol, UK) was given once a day starting 30 min after surgery until the mice were euthanized. All the compounds were dissolved in 0.9% saline solution and administered in a volume of 10 mL/kg. The dosage of Sig-1R ligands was chosen based on previous studies showing benefit in MN degeneration, ATR-X syndrome, retinopathy and pain models [24,25,54,55], and preliminary assays in our laboratory. Control animals were administered with saline.

### 4.2. Rhizotomy Surgery

Surgery was performed under anesthesia with ketamine (100 mg/kg) and xylazine (10 mg/kg) intraperitoneally. An incision was made in the skin of the dorsum above the iliac crest and a small laminectomy was performed to expose the right L4–L5 spinal roots, and the roots were cut at postganglionic level [56] (Figure 1A). Then, the stumps of the roots were separated in opposites sides to avoid axon regeneration. The incision was sutured and disinfected with povidone iodine, and the mice cared until recovery in warm environment. Analgesia was provided with buprenorphine (0.1 mg/kg s.c.) for the next 2 days. During all the follow up, the mice were checked for assessment of general health and mobility daily during the first week and later once per week.

### 4.3. Retrograde Labelling

Retrograde tracing was applied to the sciatic nerve of mice to identify the MN pools sending axons through lumbar L4–L5 spinal roots after surgery. Under anesthesia with ketamine (100 mg/kg) and xylazine (10 mg/kg) rhizotomy was performed on the right L4–L5 roots. Thereafter, the sciatic nerve was exposed at the midthigh and sectioned. The proximal stump of the nerve was soaked in retrotracer solution (1 μL) True Blue Chloride (TB, Setareh Biotech, Eugene, OR, USA) in the left side and FluoroGold (FG, Fluorochrome, Denver, CO, USA) in the right side for 45 min to allow dye internalization. Afterwards, the wound was sutured in layers and disinfected. Animals were sacrificed 7 days post tracing.

### 4.4. Electrophysiological Tests

Motor nerve conduction tests were performed at 5 dpi and at the end of follow-up in both hindlimbs. Briefly, the sciatic nerve was stimulated with small needle electrodes placed at the sciatic notch, and the CMAP of TA, GM and plantar interossei muscles recorded with microneedle electrodes [57]. All the potentials were amplified and displayed on a digital oscilloscope at settings appropriate to measure the latency to the onset and the amplitude to the negative peak. Mice were tested under anesthesia with pentobarbital (50 mg/kg i.p.) and maintained warm by means of a thermostatic heating pad.

### 4.5. Histological and Immunohistochemical Analyses

At 7, 28 and 42 dpi subgroups of mice were deeply anesthetized with pentobarbital (200 mg/kg) and transcardially perfused with 4% paraformaldehyde in phosphate buffer (PB). The lumbar spinal cord and the muscles TA and GM were harvested, post-fixed, and cryopreserved in 30% sucrose solution in PB at 4 °C. Hindlimb muscles of both sides were weighed before cryopreservation.

For assessing MN preservation, 20 µm transverse thick sections were serially cut on a cryostat (Leica, Wetzlar, Germany). Each section of a series of 10 was collected sequentially on separate slides. L4–L5 lumbar spinal cord sections of each animal separated 100 µm were rehydrated and stained for 3 h with an acidified solution of cresyl violet 3.1 mM. The slides were dehydrated with ethanol, cleared with xylol, and mounted with DPX. Images of L4–L5 lateral ventral horn stained with cresyl violet were captured at 20x (Nikon Eclipse Ni, Tokyo, Japan). Then, the number of MN per each section was counted. MNs were identified if they had diameter larger than 20 µm, polygonal shape and prominent nucleoli.

For immunohistochemistry of XBP-1, the endogenous peroxidase activity was inhibited with 70% methanol, 30% Tris-buffered saline (TBS) and 2% H2O2. Lumbar spinal cord sections were blocked with TBS with 3% Triton-X-100 and 1.5% normal donkey serum. Slides were incubated overnight at 4 °C with primary antibody against rabbit anti-XBP-1 (1:200, ab37152, Abcam, Cambridge, UK). Slides were washed with TBS with 0.1% tween 20 and incubated with a secondary antibody horse anti-rabbit biotinylated (Vector Laboratories, Burlingame, CA, USA) overnight at 4 °C. Afterwards, slides were incubated using VECTASTAIN^®^ Elite ABC complex for 1 h and DAB solution (Vector Laboratories, Burlingame, CA, USA) for brown color development. Finally, after dehydration with a series of ethanol gradients and xylol clearing, slides were mounted with DPX. Images were captured at 20× (Nikon Eclipse Ni, Tokyo, Japan).

To identify Sig-1R localization in spinal cord samples, antigen retrieval was performed by incubating samples with pre-boiled citrate buffer for 30 min at room temperature. The endogenous peroxidase and biotin activity were inhibited with Invitrogen Kit (E21390, B40933, Invitrogen, Waltham, MA, USA). Lumbar spinal cord sections were incubated with blocking solution for 1 h, then incubated overnight at 4 °C with primary antibody rabbit anti-Sigma-1 Receptor (1:100; 223702, Abcam, Cambridge, UK). Slides were washed with phosphate buffer saline (PBS) with 0.3% Triton and incubated with a secondary antibody horse anti-rabbit biotinylated (1:200, Vector Laboratories, Burlingame, CA, USA) for 1 h. After washes, samples were incubated for 1 h with streptavidin-HRP linked antibody. TSA kit was applied for 3 min for displaying AlexaFluor 555 to Sig-1R. Finally, slides were mounted with Fluoromount G (Southern Biotech, Birmingham, AL, USA). Images of the ventral horn were captured at 40× and 100× (Nikon Eclipse Ni, Tokyo, Japan).

For immunolabeling glial cells, lumbar spinal cord sections were blocked with PBS with 0.3% Triton-X-100, 10% donkey serum and 0.2 mM glycine 1 h at room temperature. Slices were then incubated overnight at 4 °C with primary antibodies rabbit anti-Iba1 (1:500; 019-19,741, Wako, Osaka, Japan), and rat anti-GFAP (1:500; 13-0300, Invitrogen, Waltham, MA, USA). After washes with PBS with 0.1% tween 20, sections were incubated for 2 h with the respective secondary antibodies: Alexa 488-conjugated secondary antibody (1:500) or Cy3-conjugated secondary antibody (1:500). FluoroNissl (1:200, 990210, Invitrogen, Waltham, MA, USA) and DAPI (1:2000; D9563, Merck-Sigma, Kenilworth, NJ, USA) added to stain MNs and nuclei, respectively, and the slides were mounted with Fluoromount G. Microphotographs of the spinal cord ventral horn were captured under the same exposure time, sensitivity, and resolution (×40) for each analyzed marker by using a fluorescence microscope (Olympus BX51, Tokyo, Japan). A total of 8 images were taken per animal and side, then analyzed. The integrated density of GFAP and Iba1 labeling was measured after defining a threshold for background correction using ImageJ software. The integrated density is the product of area and mean grey value, which is the sum of the grey values of all the pixels in the selection divided by the number of pixels.

### 4.6. Protein Extraction and Western Blot Analysis

Mice were deeply anesthetized at 7 and14 dpi (*n* = 3–5) to obtain L4–L5 spinal cord segments of control and rhizotomized mice with or without Sig1-R ligands treatment for Western blot analyses. Samples were divided in ipsi- and contralateral halves and frozen in liquid nitrogen for storage. Tissue samples were homogenized in RIPA buffer (50 mM Tris-Cl pH 7.4, 150 mM NaCl, 2 mM EDTA, 1% triton- X-100, 0.5% sodium deoxycholate, 0.1% sodium dodecyl sulfate) containing a mixture of protease inhibitor cocktail (Merck-Sigma, Kenilworth, NJ, USA) and phosphatase inhibitors (PhosphoSTOP Roche, Basilea, Switzerland). Lysates were homogenized on ice using a Potter homogenizer, sonicated, and centrifuged at 12,000 rpm during 10 min at 4 °C. Protein concentration was determined by BCA Protein Assay (Biorad, Hercules, CA, USA). For Western blotting, equal amount of protein per each sample (20–30 μg) were loaded onto 7.5–15% SDS-polyacrylamide gels and transferred in a PDVF membrane for 1.30 h at 90 V and room temperature. Membranes were blocked with 6% milk or 5% BSA (for phosphorylated antibodies) in TBS 0.1% Tween-20 for 1 h and then, incubated with primary antibodies at 4 ºC overnight. The following antibodies were used: rabbit anti-Sigma-1 Receptor (1:250; 223702, Abcam, Cambridge, UK), rabbit anti-GRP78/BiP (1:500, G8918, Merck-Sigma, Kenilworth, NJ, USA), rabbit anti-IRE (phosphor S724) (1:200, 48187, Abcam, Cambridge, UK), rabbit anti-IRE1α (1:500, 3294, Cell Signaling, Danvers, MA, USA), rabbit anti-XBP-1 (1:250; 37152, Abcam, Cambridge, UK), mouse anti-GADD153/CHOP (1:500; 3751, Santa Cruz Biotech., Dallas, TX, USA), mouse anti-actin (1:50,000; A5316, Merck-Sigma, Kenilworth, NJ, USA). The following day, after several washes, the membrane was incubated with secondary antibody HRP-conjugated (1:5000; Biorad, Hercules, CA, USA) for 1 h at room temperature. Proteins were visualized using enhanced chemiluminescence method (ECL Clarity kit, BioRad Laboratories, Hercules, CA, USA) and images were captured using Chemidoc apparatus. Image Lab software (BioRad, Hercules, CA, USA) was used for image density quantification. Levels of each protein were normalized by the housekeeping protein (ß-actin) and by each control sample.

### 4.7. Statistical Analyses

All data are expressed as mean ± SEM. Statistics were performed using GraphPad Prism 6 software. Data were analyzed using ANOVA followed by Bonferroni’s post-hoc test. For molecular analysis data were analyzed by one-way ANOVA followed by Dunnett’s post-hoc test. Differences were considered significant at *p*-value ≤ 0.05.

## 5. Conclusions

In summary, the survival of spinal MNs in adult mice following postganglionic rhizotomy is dependent on the time after the lesion, inducing a loss of about 40% of the spinal MNs at 42 days. Treatment with different Sig-1R ligands exerted neuroprotective effects, preventing cell death by maintaining the IRE1/XBP1s axis, increasing the number of surviving MNs and reducing glial reactivity in the ventral horn after rhizotomy. These and previous findings suggest that Sig-1R is a promising target for the treatment of MN degeneration.

## Data Availability

Data is available from the authors under reasonable request.
